# Identification of Human HK Genes and Gene Expression Regulation Study in Cancer from Transcriptomics Data Analysis

**DOI:** 10.1371/journal.pone.0054082

**Published:** 2013-01-31

**Authors:** Meili Chen, Jingfa Xiao, Zhang Zhang, Jingxing Liu, Jiayan Wu, Jun Yu

**Affiliations:** 1 CAS Key Laboratory of Genome Sciences and Information, Beijing Institute of Genomics, Chinese Academy of Sciences, Beijing, China; 2 Graduate University of Chinese Academy of Sciences, Beijing, China; University of Alabama at Birmingham, United States of America

## Abstract

The regulation of gene expression is essential for eukaryotes, as it drives the processes of cellular differentiation and morphogenesis, leading to the creation of different cell types in multicellular organisms. RNA-Sequencing (RNA-Seq) provides researchers with a powerful toolbox for characterization and quantification of transcriptome. Many different human tissue/cell transcriptome datasets coming from RNA-Seq technology are available on public data resource. The fundamental issue here is how to develop an effective analysis method to estimate expression pattern similarities between different tumor tissues and their corresponding normal tissues. We define the gene expression pattern from three directions: 1) expression breadth, which reflects gene expression on/off status, and mainly concerns ubiquitously expressed genes; 2) low/high or constant/variable expression genes, based on gene expression level and variation; and 3) the regulation of gene expression at the gene structure level. The cluster analysis indicates that gene expression pattern is higher related to physiological condition rather than tissue spatial distance. Two sets of human housekeeping (HK) genes are defined according to cell/tissue types, respectively. To characterize the gene expression pattern in gene expression level and variation, we firstly apply improved K-means algorithm and a gene expression variance model. We find that cancer-associated HK genes (a HK gene is specific in cancer group, while not in normal group) are expressed higher and more variable in cancer condition than in normal condition. Cancer-associated HK genes prefer to AT-rich genes, and they are enriched in cell cycle regulation related functions and constitute some cancer signatures. The expression of large genes is also avoided in cancer group. These studies will help us understand which cell type-specific patterns of gene expression differ among different cell types, and particularly for cancer.

## Introduction

Gene expression regulation contains the process that cells and viruses use to regulate the way that the information in genes is turned into gene products, most of which are protein coding genes [1]–[3]. Gene expression regulation is essential for eukaryotes [4] because it drives the processes of cellular differentiation and morphogenesis [5]. This leads to the creation of different cell types in multicellular organisms, where different cell types may possess different gene expression profiles, though they all possess the same genome sequence [6]. A major challenge in current research is how to define the mode of gene expression regulation. Based on gene expression breadth [7]–[9], genes can be divided into ubiquitously expressed genes [6]–[10], near universal HK genes, and tissue-specific/cell-specific genes. Based on the gene expression level, the gene can be determined as a low/high expression gene [11], and as a constant/variable expression gene [12]–[13]. Gene structure is one important regulation factor for gene expression. It is comprised mainly of gene structure composition, gene structure organization, gene variation, protein classes, cellular structure, cellular processes, and molecular mechanisms [10], [14]–[25].

RNA-Seq is becoming a more and more popular biotechnology because of its transcription measurement at predominant precision and high-throughput to detect weakly expressed genes [10]–[11], [15], [26]. Due to the dramatic advances in RNA-Seq, transcriptome data increase rapidly [25]–[27] in SRA database. In previous cancer progression and gene expression regulation mechanisms studies based on microarray data [28]–[30], researchers mainly compared gene expression in cancer condition vs. normal condition with the same originals. This method could miss many truly up-regulated different expression (DE) genes by the normalization process [31], disregarding the based mechanism in cancer. In this study, we select 12 normal samples and 9 cancer samples to explore the general mechanism of cancer gene expression regulation from RNA-Seq transcriptome data. We define the gene expression pattern from three directions and characterize cancer HK genes to observe gene expression regulation in cancer cells. This research will help us understand the key regulatory genes and the pathogenesis of cancer.

## Materials and Methods

### RNA-Seq transcriptome dataset

RNA-Seq samples under normal and cancer condition are selected to identifying HK genes. Two major elements are considered for the selection, the amount and saturation of the selected samples. Although RNA-Seq samples are voluminous in the public data resource, the useful samples for normal vs cancer comparative analysis are limited. If we had included more unsaturated samples, it would have lead to a higher false negative rate mainly caused by low abundance genes. We totally obtain 37 different human tissue/cell line transcriptomics data from public SRA database (Table S1), 22 normal samples and 15 cancer samples. Then we select samples with criterions as follows: 1) removing all mixed cell lines samples, because pooling method will cover differential gene expression abundance; 2) removing cell lines samples with special treatment, because regulation mechanisms are different under diverse physiological conditions; 3) filtering severe unsaturated datasets; 4) selecting the most saturated sample if replicates existed, we do not prefer integration which would induce higher false negative rate; 5) selecting samples coming from Illumina Genome Analyzer, the most popular sequencing instrument, here we try to reduce the original difference between various sequencing platforms. Finally, we get 12 normal tissues and 9 cancer cell lines for further analysis. The normal tissues in our analysis include adipose, brain, cerebral cortex, colon, breast, kidney, liver, lung, lymph node, heart, testes, and skeletal muscles. And cancer cell lines include K562, DLD-1, HepG2, GM12878, Lymphoma, BT474, MCF7, MB435, and T47D in current RNA-Seq datasets (Table S1). K562 is an immortalized cell line produced from a female patient with chronic myelogenous leukemia (CML). DLD-1 is a colon adenocarcinoma cell line cultured under 21% oxygen with non-targeting siRNA transfected. HepG2 is a cell line derived from a male patient with liver carcinoma. GM12878 is a lymphoblastoid cell line produced from the blood of a female donor by EBV transformation. Lymphoma is a Ramos B cell. The other cell lines are all breast cancer cell lines derived from invasive ductal carcinomas (ATCC). MCF-7, BT474 and T47D are oestrogen-receptor-positive and progesterone-receptor-positive; MD435 is negative for both. High quality CEL files of human microarray data on HG-U133A are selected from AffayExpress (E-MTAB-27) [32] (Table S2) for the comparison.

After random transcripts filtering, we select 28,778 human RefSeq protein coding transcripts (RefGene of UCSC annotation database, Jan 4, 2010 update), and cluster them into 18,874 human loci as described previously [9]. 13,038 (69.08%) genes with multi-isoforms and 5,836 (30.92%) genes with single-isoform are used for further analysis. To map transcriptional data sets onto their reference genomic sequence GRCH37 (hg19), we use MAQ mapping software [33] downloaded from UCSC. Then annotation of the mapping results is compared to RefGene.

### The transcriptome data analysis model

Gene expression abundance is normalized as read density, i.e., reads per kilobase (KB) of coding sequence (CDS) per million reads (RPKM), in RNA-seq data that one million mappable reads in one experiment [34]. And the expression of one gene is defined as the sum of expressions of all isoforms that belong to that gene [11]. To compute a gene expression level accurately, we cite a Poisson distribution model to estimate isoforms expression [11]. Considering time cost, we strictly require a read falls into an exon with neglecting exon-junction information.

To determine whether a gene is expressed or not, the background threshold value of gene expression is performed using a previous method that coordinated false positive rate (*FPR*) and false negative rate (*FNR*) [10]. In this paper, we define positive set as genes with reads fall into its exons, and negative set as genes with reads fall into intergenic regions. An observed expression value, which is larger than the background threshold is marked as positive, and the opposite is marked as negative. Then, we get these two definitions, 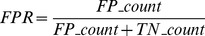, 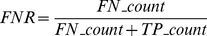 (*FP_count* means the summary of intergenic region counts for expression value larger than background, contrarily as *TN_count*. *FN_count* means the summary of gene count as gene expresses, but expression value smaller than background, conversely as *TP_count*).

Identification of low and high expression genes can depict gene expression pattern in a sample, and dynamical alteration of gene expression level among tissues/cell lines reflect the inner reaction of gene expression regulation. Previous studies usually divided gene expression level into several intervals, and marked two extreme genes as low and high, respectively [11]. This definition is somehow arbitrary, because it measured gene expression level regardless of gene expression pattern. Meanwhile, expression level discrepancy of adjacent expression level genes in two sequential subgroups might be weakly. Driven by this motivation, we firstly apply the improved K-means algorithm to identify low and high expression thresholds dynamically, which divide expressed genes into three categories: low expression genes (LEG), moderate expression genes (MEG), and high expression genes (HEG). As to one sample, low expression threshold is defined as the average value of maximum gene expression value in LEG and minimum gene expression value in MEG. In order to analysis the gene expression pattern variation among different samples, we define a unified low expression threshold as the median value of all samples' low expression thresholds. High expression threshold for one sample is defined as the average value of maximum gene expression value in MEG and minimum gene expression values in HEG. And the unified high expression threshold is the median value of all samples. The method is based on individual gene expression distribution pattern of a sample to identify low and high expression genes with dynamical measurement. And it guarantees the maximum distance of gene expression level of two sequential subgroups.

The improved K-means algorithm assigns each expressed genes to the cluster whose centroid is nearest as K-means algorithm do. But distance of two elements is defined as absolute value of difference of two gene expression values. Centroid is defined as expression value of the middle gene in the cluster of sorting genes according to gene expression value. That is different from K-means algorithm defined as arithmetic mean. We initialize gene expression dataset into a point format (*x*, *y*), where *x* is gene expression value and y is its corresponding gene count. The algorithm is roughly described as follows:

1. Transform *x* value by the formula 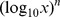, where *n* is transform factor and its default value is 1.
2. Set the number of cluster *K* ( = 3).
3. Randomly select *K* elements from point set as centroids of clusters.
4. Assign each point to the nearest cluster centroid.
5. Re-compute *K* new cluster centroids.
6. Go to 4) until the assignment has not changed any more.

As a result, expressed genes are divided into 3 categories: LEG, MEG, and HEG. We set normal group results as the control standard. The median values of low thresholds and high thresholds in 12 normal tissues are set as finally low threshold and high threshold for all tissues/cell lines.

We use the variance of gene expression level to depict gene expression variation, as previous studies did [35]–[37]. High expression values, which may amplify variation, contribute to variance more directly, while small values of gene expression affect variance weaker, which may conceal real variation. Thus, gene expression values are ranked as 1, 2, or 3, to represent the gene expression level as low, moderate, or high, respectively. We use these representations instead of gene raw expression value to estimate the gene expression variation pattern. For any gene, we calculate coefficient of variation value (*CV*) based on gene expression rank, 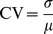, where *μ* is arithmetic average of gene expression ranks of all tissue/cell line samples in a gene; *σ* is standard deviation of gene expression rank in a gene, which is the arithmetic mean of the squared deviation of gene expression rank from its arithmetic mean. We also set normal group as the control.

We propose an MDAD plot to characterize the discrepancy of gene expression pattern in cancer condition vs. normal condition, based on the widely used MA plot. M Distance (MD) and A distance (AD) of any gene in MDAD plot are defined as 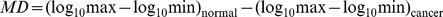 and 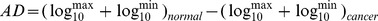, respectively, where *max* value in 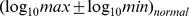 is the maximum gene expression value within all normal tissue/cell line samples, and *min* value in 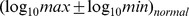 is the minimum gene expression (but >0) within all normal tissue/cell line samples; *max* value in 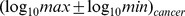 is the maximum gene expression value within all cancer tissue/cell line samples, and *min* value in 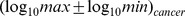 is the minimum gene expression value (but >0) within all cancer tissue/cell line samples. *MD* reflects the difference of gene expression distribution between cancer condition and normal condition, and *AD* reflects the difference of relative average level between cancer condition and normal condition. We use MDAD plot, with a paired Wilcoxon signed-rank test [38], to compare the difference of shared or cancer-associated HK gene expression level between normal and cancer condition. *MD*<0 means the gene expression distribution in cancer condition is wider than that in normal condition, and *AD*<0 means the gene expression relative average level in cancer condition is higher than that in normal condition. To compare their original maximum and minimum expression levels under cancer and normal condition, we also calculate *maxR* and *minR* as the ratio of maximum and minimum expression value in normal vs cancer codintion (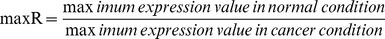, 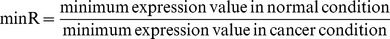). If a ratio value is 0, a gene only turn on in cancer condition; if a ratio value locates at [0, 1], extreme expression value in normal condition is smaller than that in cancer condition, if a ratio value locates at [1, ∞], extreme expression value in normal tissues is larger than that in cancer condition.

The Spearman correlation of gene expression profile is used to define the expression pattern similarity of different tissues/cells. Based on their degree of similarity, a hierarchical cluster with correlation information is conducted using R software. Normalization of microarray data use MAS5.0 [39] algorithm with Expression Console™ software (detection p-value as 0.05). Function enrichment analysis of different HK genes types is performed with David (Database for Annotation, Visualization, and Integrated Discovery) [40].

## Results

### Analysis model for RNA-Seq transcriptome data

RNA-Seq has powerful ability to detect low abundance transcripts with unprecedented accuracy and high-throughput at a much lower cost comprising with other methods. Now it has become the most widely used transcriptomics sequencing technology [11], [41]. A common query in RNA-Seq data analysis is how to define the number of expressed genes in one sample. To eliminate contamination and error caused by experiments and instruments, etc., we detect the expression level between exons and intergenic regions to coordinate *FPR* and *FNR* (see Materials and Methods section) using the method generated in a previous study [10]. The background thresholds of gene expression for individual samples are falling in 0.13–0.41 RPKM. We set a median value of 0.25 RPKM (Figure S1) as the background threshold of gene expression for further analysis. Then we use a Poisson model to deal with isoform expression estimation and refine the gene expression value by accumulating all isoforms expression values in one gene [11].

### Definition of HK genes

**Figure 1:**
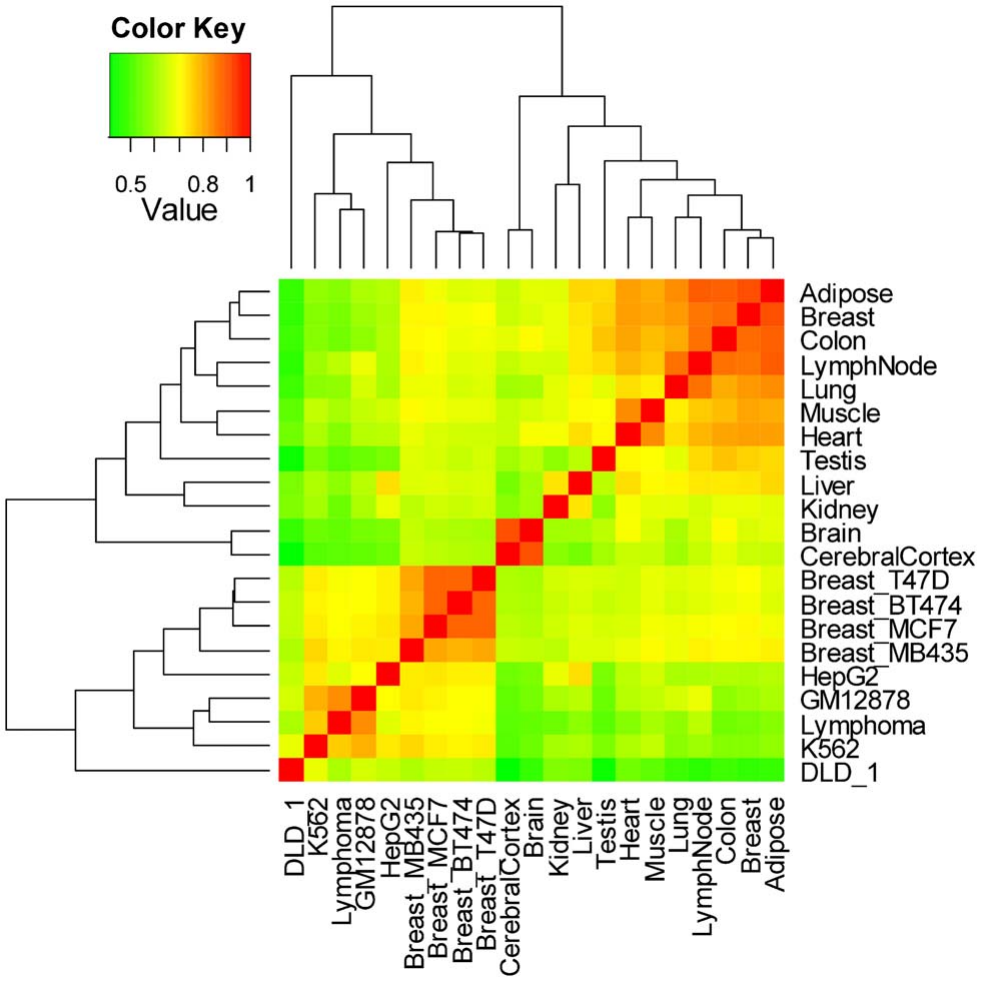
Gene expression profiles hierarchical cluster analysis based on Spearman correlation. Spearman correlation of gene expression profiles is used to define the gene expression profiles similarity of 21 different tissues/cells. A hierarchical cluster analysis with correlation information shows 2 clusters: 12 normal tissues and 9 cancer cell lines.

Our samples are separated into two physiological groups: 12 normal tissues and 9 cancer cell lines, details are shown in Table 1. The cluster analysis indicates that gene expression patterns are highly related to physiological condition rather than tissue spatial distance (Figure 1). We predict that there are some common regulation patterns in cancer cells, such as turn on/off regulation and low/high or constant/variable adjustment, which maintain their limitless proliferation ability. Here, we define HK genes in two separate groups, normal HK genes and cancer HK genes, to reflect gene expression on/off status in different physiological condition. Previous study on hierarchical clustering of nine lung SAGE libraries also showed a clear separation of tumor and normal samples [42].

**Table 1:** RNA-Seq sample source.

Sample	Physiological	Source	Reads number	Read length
Adipose	Normal	Wang et al. 2008	22,623,849	32
Brain	Normal	Wang et al. 2008	8,168,675	32
Breast	Normal	Wang et al. 2008	13,605,904	32
Colon	Normal	Wang et al. 2008	22,696,019	32
Heart	Normal	Wang et al. 2008	17,057,207	32
Liver	Normal	Wang et al. 2008	30,654,338	32
Lymph Node	Normal	Wang et al. 2008	23,063,505	32
Muscle	Normal	Wang et al. 2008	132,812,008	32
Testis	Normal	Wang et al. 2008	24,157,076	32
BT474	Cancer	Wang et al. 2008	15,406,197	32
T47D	Cancer	Wang et al. 2008	13,871,105	32
MB435	Cancer	Wang et al. 2008	16,301,833	32
MCF7	Cancer	Wang et al. 2008	13,963,854	32
Cerebral Cortex	Normal	Pan et al. 2008	26,327,918	32
Lung	Normal	Pan et al. 2008	21,650,253	32
Kidney	Normal	Marioni et al. 2008	14,568,451	36
Lymphoma	Cancer	Sultan et al.2008	2,493,234	27
DLD-1	Cancer	Tsuchihara et al. 2009	4,542,765	36
HepG2	Cancer	ENCODE project 2008	13,646,471	33
K562	Cancer	ENCODE project 2008	21,778,871	33
GM12878	Cancer	ENCODE project2008	16,451,652	33

We define five types of HK genes according to their gene expression pattern in normal and/or cancer condition: 1) normal-unique HK genes, specific HK gene only shown in normal group, not HK gene in cancer group; 2) cancer-associated HK genes, specific HK gene only shown in cancer group, not HK gene in normal group; 3) share HK genes, HK genes expressed in both normal and cancer group; 4) normal HK genes, HK genes expressed in the whole normal group, includes normal-unique HK genes and share HK genes; 5) cancer HK genes, HK genes expressed in the whole cancer group, includes cancer-associated HK genes and share HK genes.

As to the normal group, 12 selected normal tissues cover connective tissue, muscle tissue, body region and 6 human taxonomy systems, including urogenital system, digestive system, respiratory system, hemic and immune systems, central nervous system, and cardiovascular system (Endocrine system was not covered, Figure S2). Based on these 12 normal tissues, we estimate that there are 8831 normal HK genes (protein-coding HK genes).The HK gene fraction is 47%, which is consistent with two previous reports: 40% [9] and 42% [10]. The latter investigation was also carried out with RNA-Seq data, but Daniel Ramsköld and his coworkers defined HK genes without distinguishing normal or cancer group. 8041 HK genes were identified by 24 human tissues/cell lines (10 normal tissues and 4 cancer cell lines are also considered in our study), including 7695 protein-coding genes, 277 lncR, and 69 unknown genes not present in the reference genomic sequence GRCH37, hg19 [10]. The HK genes overlap between Daniel Ramsköld *et al.*'s work and our normal HK genes are 7004 (Figure S3). And the unique HK gene in our definition (1827) mainly comes from normal-unique HK gene (1253), which is only shown as HK genes in normal condition. Since Daniel Ramsköld and his coworkers used 4 cancer cell lines, this difference of HK gene identification occurs in our study is fairly reasonable. Most of our defined 8831 normal HK genes are ubiquitously expressed in all 19 available normal samples, 12 of them are selected for normal HK gene definition, 7 of them are filtered by criterions shown in Materials and Methods (Figure S4A, Table S1). The “false detection rate” is mainly caused by unsaturation of the filtered samples. It means that the accuracy of HK genes defined from 12 normal tissues is high enough for further analysis.

**Figure 2:**
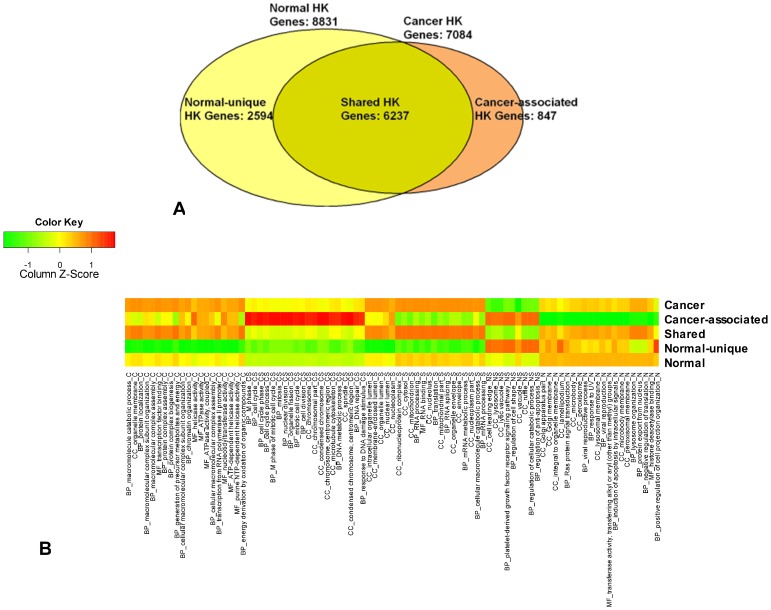
(A) Comparison of normal and cancer HK genes definition. HK genes are defined separately from two physiological groups: 12 normal tissues and 9 cancer cell lines. **(B) Different HK gene types functional enrichment.** “Cancer” means cancer HK genes, abbreviated as suffix “C” follows function term illustration; “Cancer-associated” means specific HK genes in cancer condition, abbreviated as suffix “CA” follows function term illustration; “Shared” means overlapped HK genes in normal and cancer conditions, abbreviated as suffix “S” follows function term illustration; “Normal-unique” means specific HK genes in normal condition, abbreviated as suffix “NU” follows function term illustration; “Normal” means normal HK genes, abbreviated as suffix “N” follows function term illustration.

Current cancer samples represent body region and three widely investigated human taxonomic systems, including: urogenital system, digestive system, and hemic and immune systems (Figure S2, Table S1). Our selected 9 cancer cell lines cover most of them, except the urogenital system sample, which is filtered by the unsaturation and platform selection criterions. The fraction of cancer HK gene is 38% in gene expression breadth of 9. We defined 7084 cancer HK genes and most of them are present in normal group (Figure 2A), which forms the shared HK group. Those shared 6237 HK genes could be essential genes for a cell, which maintain basic functions in different physiological condition. Cancer HK genes are less than normal HK genes because cancer required less turned on genes (Table S1). But cancer required a higher fraction of mRNA pool [10], [26] to reduce cancer cell transcriptome specialization [26]. This allows a focus on completion of simple cell proliferation. About 88.65% of cancer HK genes are ubiquitously expressed in all 13 cancer samples, including 4 filtered samples (Table S1, Figure S4B). The “false detection rate” of cancer HK genes is mainly caused by the unsaturation of the filtered samples. This result indicates that although the current 9 cancer samples can't represent various cancer types, the identification of cancer HK genes can be used in gene expression pattern study of cancer cell.

An HK gene is typically a constitutive gene that is required for the maintenance of basic cellular function, and it is found in nearly all human cells [7], [43]. To characterize normal and cancer HK gene functions, we compare cell gene function enrichment and signal pathways. As Figure 2B shows, cancer HK genes are enriched in molecular function and biological processes. Cancer HK genes participate in cell cycle, DNA replication, mismatch repair, and apoptosis pathway, etc., to reply to tumor occurrence. Normal HK genes tend to join in basic pathways (Table 2).

**Table 2:** Functional enrichment analysis of normal and cancer HK genes.

Gene Type	KEGG pathway	Benjamini^1^	Percent(%)^2^	Gene count
Normal HK Genes	Adherens junction	7.61E-5	0.68	60
	Pyruvate metabolism	4.06E-3	0.37	32
	Propanoate metabolism	9.67E-3	0.30	26
Cancer HK genes	Cell cycle	1.96E-17	1.42	100
	Pyrimidine metabolism	8.34E-14	1.10	77
	Alzheimer's disease	1.21E-10	1.56	110
	Parkinson's disease	3.99E-10	1.28	90
	DNA replication	3.31E-8	0.47	33
	Oocyte meiosis	1.90E-5	1.00	70
	Neurotrophin signaling pathway	2.00E-5	1.10	77
	Mismatch repair	5.56E-5	0.30	21
	Apoptosis	2.95E-4	0.78	55
	SNARE interactions in vesicular transport	9.52E-4	0.40	28
	Pancreatic cancer	9.52E-4	0.65	46
	Homologoesus recombination	1.50E-3	0.31	22
	Acute myeloid leukemia	1.80E-3	0.54	38
	Vibrio cholerae infection	3.94E-3	0.51	36

1. Benjamini value is a globally correct enrichment p-values to control family-wide false discovery rate under certain rate (e.g., ≤0.05). It is one of the multiple testing correction techniques (Bonferroni, Benjamini, and FDR) provided by DAVID.

2. The percentage of normal or cancer HK genes participate in a pathway.

### Characterization of shared HK genes expression patterns

To characterize gene expression level and variation leading to gene expression patterns definition, we firstly apply improved K-means algorithm and adopt improved gene expression coefficients of variance (*CV*, see Materials and Methods for details) model. Previous studies usually defined 100 RPKM genes as high expression threshold values and the 1 RPKM for low expression based on eight log-scale bins [11]. The improved K-means algorithm identifies thresholds from an individual gene expression distribution pattern. Based on the calculation of this algorithm, low expression threshold values are 0.66–1.22 RPKM, and high expression threshold values are 8.58–19.99 RPKM (Table 3). We set a median value of 1.06 RPKM for low threshold and a median value of 12.72 RPKM for high threshold in normal condition as a standard for further analysis (Figure S5). To discriminate a gene expression variation status, we apply an improved gene expression *CV* model. The *CV* values in normal group range from 0 to 0.54. Q1 (one quarter) and Q3 (three quarters) *CV* values in normal group are 0.14 and 0.26, which are marked as constant and variable expression threshold values, respectively (Figure S6). Thus, we totally get three statuses of gene expression variation, constant (0<*CV*≤0.14), moderate variable (0.14<*CV*≤0.26), and variable (*CV*>0.26).

**Table 3:** Low and high gene expression thresholds calculated by the improved K-means algorithm.

Sample	Low expression			High expression		Total gene
	Gene count	Threshold	Moderate	Gene count	Threshold	
Adipose	3,101	1.20	8,505	1,313	19.99	12,919
Brain	2,949	1.22	8,903	1,553	13.14	13,405
Breast	3,311	1.18	8,985	1,289	17.66	13,585
Cerebral Cortex	3,385	0.66	9,094	1,522	9.47	14,001
Colon	3,402	1.18	8,570	1,300	18.00	13,272
Heart	2,855	0.88	7,950	1,386	9.99	12,191
Kidney	2,868	0.80	8,991	1,710	8.58	13,695
Liver	2,895	0.85	7,679	1,509	12.30	12,083
Lung	3,399	0.71	9,111	1,482	9.99	13,992
Lymph Node	3,229	1.19	8,994	1,656	14.43	13,879
Muscle	4,769	1.18	5,872	1,072	18.69	11,713
Testis	2,538	0.93	10,425	3,004	11.75	15,967

**Figure 3:**
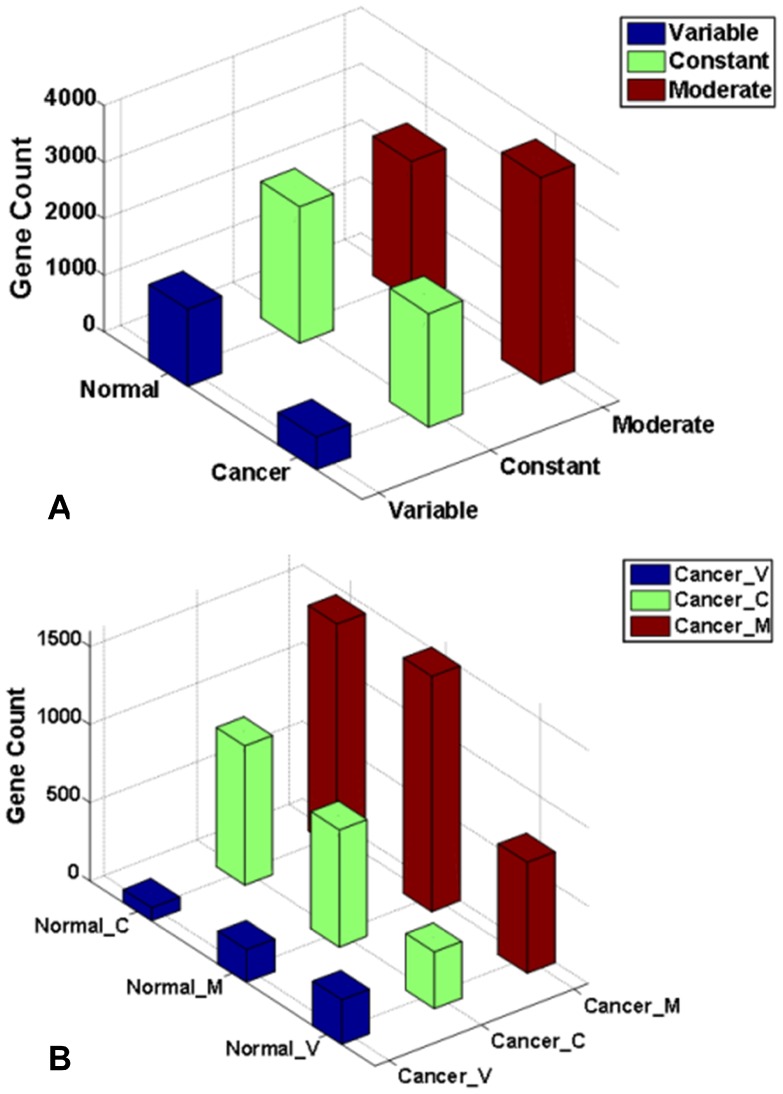
(A) Shared HK genes expression variation distribution in normal and cancer condition. (B) Gene expression variation adjustment in shared HK genes between normal and cancer condition. There are three gene expression variation statuses, Constant, abbreviated as suffix “C” in (B), and Moderate variable, abbreviated as Moderate in (A) and suffix “M” in (B), and Variable, abbreviated as suffix “V” in (B).

It is well known that some genes express constantly among tissues while others express variably in normal condition. This phenomenon also exists in HK genes [12]–[13], [35]. Based on gene expression *CV* model, we find that more HK genes in cancer tend to be moderate variable expressed genes (Figure 3A). We attempt to investigate the ways in which gene expression variation status is regulated to deal with the emergence of a tumor. Thus, we compare 6237 shared HK genes to illustrate their adjustment. More than one half of shared HK genes' expression variation status changes between normal and cancer condition. As shown in Figure 3B, nearly two-thirds of constant shared HK genes under normal condition change to moderate variable status under cancer condition. One third of moderate variable shared HK genes in normal condition become constant shared HK genes in cancer condition. About one half of variable shared HK genes in normal condition change their expression variation status to moderate variable in cancer condition (Figure 3B). A cell is apt to modulate its gene expression pattern to be mainly moderate variable expression in tumor physiological condition.

**Figure 4:**
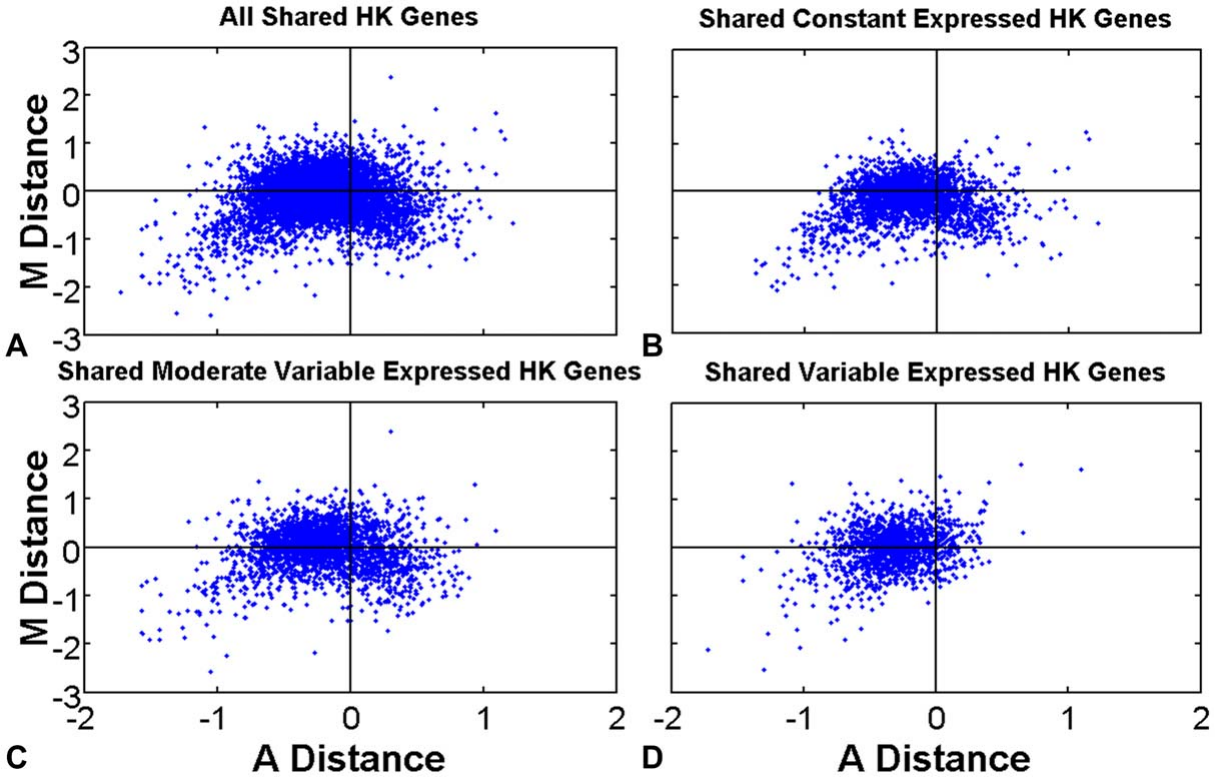
MDAD plots of shared HK genes. *MD*<0 means the gene expression span width in cancer condition is larger than that in normal condition, and *AD*<0 means the gene expression relative average level in cancer condition is higher than that in normal condition. According to shared-normal HK genes expression variation statuses, shared HK genes are divided into three subtypes, constant, moderate variable, and variable expressed shared HK genes. Paired Wilcoxon signed rank test is used here to measure gene expression regulation and gene expression variation status regulation. (A) All shared HK genes. (B) Shared constant expressed HK genes. (C) Shared moderate variable expressed HK genes. (D) Shared variable expressed HK genes.

To measure gene expression regulation and gene expression variation status regulation in cancer condition, we propose an MDAD (see Materials and Methods section) plot with a paired Wilcoxon signed-rank test [38] in all shared HK genes (Figure 4A) and shared HK genes in three variation status subtypes (Figure 4B, C, D). All paired Wilcoxon signed-rank test detail values are shown in Table 4. Shared HK genes express higher in cancer than in normal tissues, based on the effective expression width (*MD*, p-value is 4.34E-33) and the intermediate value (*AD*, p-value is 0). The previous microarray data indicated that human cancer genes may be widely up-regulated [31]. Paired Wilcoxon signed-rank test p-values of *MD* in the three gene expression variation subtypes are 4.24E-67, 0.11, and 0.59, respectively. P-values of *AD* are all too lower with the values of 3.15E-160, 2.62E-126, and 3.65E-183 (Table 4). As Figure 4 shown, most shared HK genes' *AD* and *MD* values are smaller than 0 which means genes express higher in cancer condition than in normal condition. Thus, in cancer condition, a cell mainly adjusts constant shared HK genes to express higher to act the emergence of cancer signal.

**Table 4:** Comparison of paired Wilcoxon signed-rank test results of MDAD values for shared HK genes in normal and cancer condition.

Type^1^	Gene count	Paired Wilcoxon signed rank test result of null hypothesis^2^	P-value	Paired Wilcoxon signed rank test statistical Z value
MD	6,237	1	4.34E-33	−11.98
AD	6,237	1	0	−45.58
MD_constant	2,417	1	4.24E-67	−17.31
AD_constant	2,417	1	3.15E-160	−26.97
MD_moderate	2,464	0	0.11	−1.62
AD_moderate	2,464	1	2.62E-126	−23.91
MD_variable	1,356	0	0.59	−0.54
AD_variable	1,356	1	3.65E-183	−28.86

1. To characterize the discrepancy of gene expression pattern in cancer condition vs. normal condition. *MD*, M Distance, is defined as 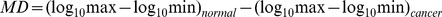; *AD*, A Distance, is defined as 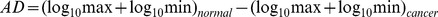. All shared HK genes are divided into three subtypes, constant, moderate variable (Moderate), and variable.

2. Null hypothesis is the dataset coming from a distribution whose median (and mean) is zero.

**Figure 5:**
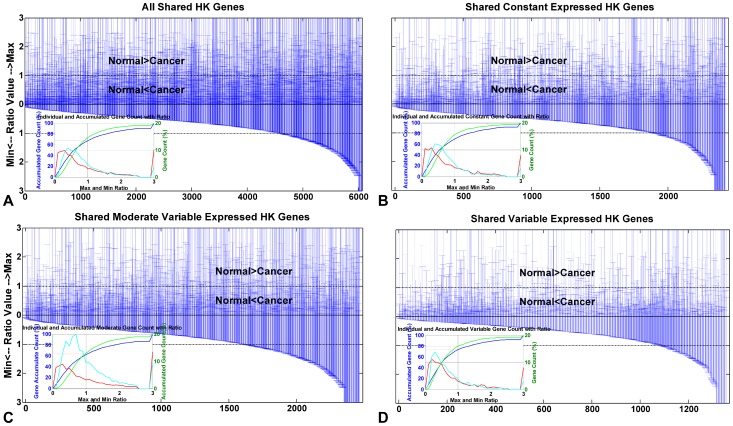
*MaxR* and *minR* value distributions of shared HK genes. Up y-axis denotes *maxR* with range [0, 3], and down y-axis denotes *minR* with range [0, 3]. For amplifying the figure, we set the ratio value as 3.00 if a ratio value is larger than 2.50. As to the inner insert graph, the blue curve shows accumulated *maxR*; and the green curve shows accumulated *minR*. Both correspond to left y-axis signifying accumulated gene count. Right y-axis denotes individual gene count (shown as Gene Count Ratio), which corresponds to a red *maxR* distribution curve and a cyan *minR* distribution curve. We quantify the proportion of genes for which cancer cell modulate gene expression level to be higher than that in normal physiological status. (A) All shared HK genes. (B) Shared constant expressed HK genes. (C) Shared moderate variable expressed HK genes. (D) Shared variable expressed HK genes.

We quantify the proportion of genes for which cancer cell modulate gene expression level to be higher than that in normal physiological status. To do so, we calculate gene counts that have maximum ratio values (*maxR*) and minimum ratio values (*minR*) ≤1 (see Materials and Methods section). When *minR*≤1, there are 73.47% of shared HK genes accumulated; when *maxR*≤1, there are 67.79% of shared HK genes accumulated (Figure 5A, Table 5). We also consider cells regulate gene expression levels in cancer condition combining with gene expression variation information. When *minR*≤1, there are 78.24% of shared HK genes in constant status, 65.10% of shared HK genes in moderate variable status, and 80.16% of shared HK genes in variable status are accumulated. And when *maxR*≤1, those number are 70.17%, 62.30%, and 73.53% in these three expression variation subtypes (Figure 5B, C, D, Table 5). The data show that most shared HK genes are up regulated combining with gene expression variation status in cancer condition.

**Table 5:** Accumulated shared HK genes ratio when *minR* and *maxR*≤1.

Type	Gene count	*minR*≤1^1^ (%)	*maxR*≤1^2^ (%)
All	6,237	73.47	67.79
Constant	1,518	78.24	70.17
Moderate	3,812	65.10	62.30
Variable	907	80.16	73.53

1. Accumulated shared HK genes ratio when *minR*≤1 (see *minR* definition in Materials and Methods section).

2. Accumulated shared HK genes ratio when *maxR*≤1 (see *maxR* definition in Materials and Methods section).

### Characterization of cancer-associated HK genes expression signatures

**Figure 6:**
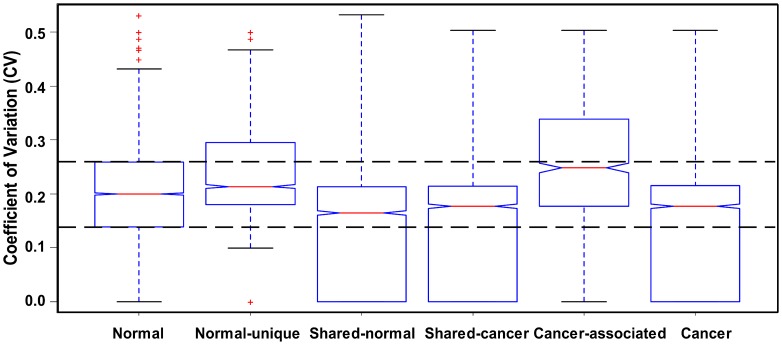
Six types of HK genes Coefficient of Variation (*CV*) values distributions. The up and down bars signify Q1 (one quarter) and Q3 (three quarters) of *CV* values, marked as constant and variable expression threshold values. “Normal” *CV* values distribution for normal HK genes; “Normal-unique” is *CV* values distribution calculated from specific HK genes in normal condition; “Shared-normal” is *CV* values distribution in 9 cancer cell lines calculated from overlapped HK genes in normal and cancer conditions; “Shared-cancer” is *CV* values distribution in 12 normal tissues calculated from overlapped HK genes in normal and cancer conditions; “Cancer-associated” is *CV* values distribution calculated from specific HK genes in cancer condition; “Cancer” is *CV* values distribution for cancer HK genes.

**Figure 7:**
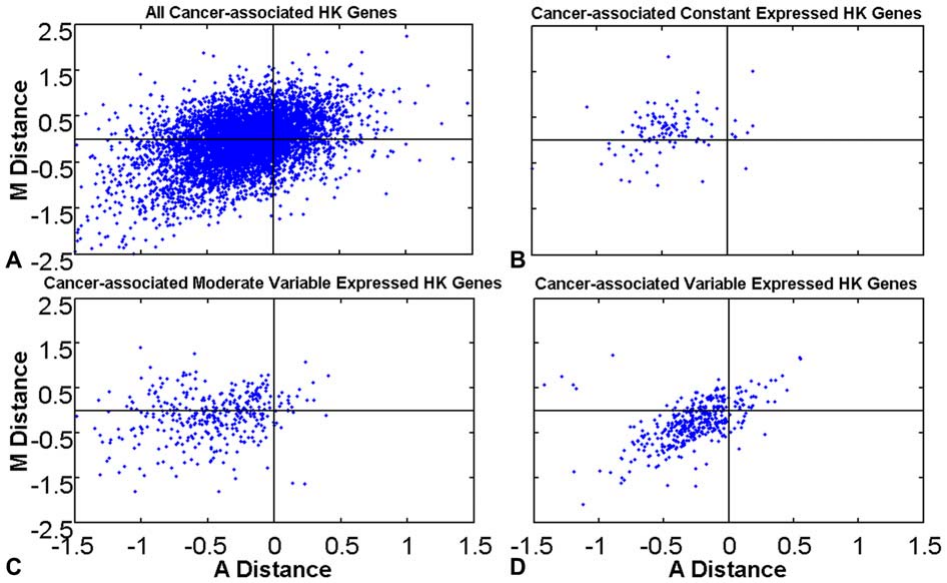
MDAD plots of cancer-associated HK genes. *MD*<0 means the gene expression span width in cancer condition is larger than that in normal condition, and *AD*<0 means the gene expression relative average level in cancer condition is higher than that in normal condition. According to cancer-associated HK genes expression variation statuses, cancer-associated HK genes are divided into three subtypes, constant, moderate variable, and variable expressed cancer-associated HK genes. Paired Wilcoxon signed-rank test is used here to measure gene expression regulation and gene expression variation status regulation in cancer. (A) All cancer-associated HK genes. (B) Cancer-associated constant expressed HK genes. (C) Cancer-associated moderate variable expressed HK genes. (D) Cancer-associated variable expressed HK genes.

**Figure 8:**
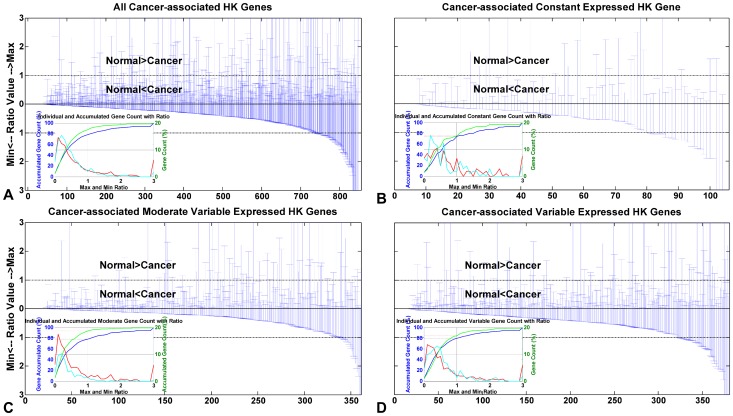
*MaxR* and *minR* value distributions of cancer-associated HK genes. Up y-axis denotes *maxR* with range [0, 3], and down y-axis denotes *minR* with range [0, 3]. For amplifying the figure, we set the ratio value as 3.00 if a ratio value is larger than 2.50. As to the inner insert graph, the blue curve shows accumulated *maxR*; and the green curve shows accumulated *minR*. Both correspond to left y-axis signifying accumulated gene count. Right y-axis denotes individual gene count (shown as Gene Count), which corresponds to a red *maxR* distribution curve and a cyan *minR* distribution curve. We quantify the proportion of genes for which cancer cell modulate gene expression level to be higher than that in normal physiological status. (A) All cancer-associated HK genes. (B) Cancer-associated constant expressed HK genes. (C) Cancer-associated moderate variable expressed HK genes. (D) Cancer-associated variable expressed HK genes.

There are only 847 cancer-associated HK genes, while there are 2594 normal-unique HK genes (Figure 2A). Normal-unique HK genes and cancer-associated HK genes undertake the basic function reacting to physiological condition, which prefer to express more variable expressed genes in three expression variation status, compared to normal (the standard control) and cancer HK genes (Figure 6). Cancer tends to turn off constant expression genes. We are interested in whether cancer regulates cancer-associated HK gene expression levels similarly to the way that shared HK gene expression levels are regulated. We do another set of MDAD plots (Figure 7) and calculate *maxR* and *minR* (Figure 8) as described above to illustrate how cancer regulates cancer-associated HK genes in gene expression levels with variation status.

Shown as Figure 7, we find that cancer-associated HK genes express higher than these genes do in normal tissues, which can be certificated by the intermediate value and the effective expression width. Most genes' *MD* values are negative value (p-value is 4.59E-15), as well as *AD* values (p-value is nearly 0, Table 6). *MD* p-values of the three gene expression variation subtypes are 1.54E-6, 1.61E-4, and 2.84E-25, respectively. While *AD* p-values are much lower with the values of 2.96E-18, 8.98E-56, and 2.08E-48 (Table 6). The paired Wilcoxon signed-rank test statistical values of *MD* and *AD* show that tumor induces cancer-associated HK genes to express higher in cancer condition than they do in normal condition.

**Table 6:** Comparison of paired Wilcoxon signed-rank test results of MDAD values for cancer-associated HK genes in normal and cancer condition.

Type^1^	Gene count	Paired Wilcoxon signed rank test result of null hypothesis^2^	P-value	Paired Wilcoxon signed rank test statistical Z value
MD	847	1	4.59E-15	−7.84
AD	847	1	0	−46.72
MD_constant	105	1	1.54E-6	−4.81
AD_constant	105	1	2.96E-18	−8.71
MD_moderate	361	1	1.61E-4	−3.77
AD_moderate	361	1	8.98E-56	−15.73
MD_variable	381	1	2.74E-25	−10.39
AD_variable	381	1	2.08E-48	−14.62

1. To characterize the discrepancy of gene expression pattern in cancer condition vs. normal condition. *MD*, M Distance, is defined as 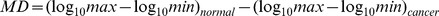; *AD*, A Distance, is defined as 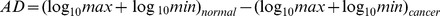. All cancer-associated HK genes are divided into three subtypes, constant, moderate variable (Moderate), and variable.

2. Null hypothesis is the dataset coming from a distribution whose median (and mean) is zero.

By analyzing *maxR* and *minR*, we find that most cancer-associated HK genes are corresponding to the *maxR*≤1 and *minR*≤1; cancer emergence actives genes express higher than they do in normal physiological condition. There are 78.51% cancer-associated HK genes with *maxR* ≤1 and 87.25% with *minR*≤1, which are shown in the accumulated curve and list (Figure 8A, Table 7). Similarly, for most cancer-associated HK genes in three expression variation subtypes, *maxR* (∼80%) and *minR* (∼90%) are ≤1 (Figure 8B, C, D). This is quite consistent with shared HK genes. So we can conclude that cancer widely up-regulate gene expression and turn off constant expression genes, while there is a bias to turn on moderate variable and variable expression status genes in cancer physiological condition.

**Table 7:** Accumulated cancer-associated HK genes ratio when *minR* and *maxR*≤1.

Type	Gene count	minR≤1^1^ (%)	maxR≤1^2^ (%)
All	847	87.25	78.51
Constant	105	75.24	74.29
Moderate	361	93.63	78.39
Variable	381	84.51	79.79

1. Accumulated cancer-associated HK genes ratio when *minR*≤1 (see *minR* definition in Materials and Methods section).

2. Accumulated cancer-associated HK genes ratio when *maxR*≤1 (see *maxR* definition in Materials and Methods section.

## Discussion

### Gene expression pattern is higher related to physiological condition rather than tissue spatial distance

In this study, the gene expression profiles of heart and muscle are similar, and cell lines form a cluster (Figure 1), which is consistent with a previous study [44]. The similar tissue types under the same physiological condition have a high profile correlation, like brain and cerebral cortex, and 4 kinds of breast tumor cell lines (Figure 1). But the same originals in two physiological conditions belong to different groups (we call them normal group and cancer group). For example, normal breast tissue and 4 kinds of corresponding tumor cells belong to normal and cancer groups, respectively (Figure 1). Due to our profile cluster result, normal and cancer samples clearly cluster into two obvious groups (Figure 1). This means gene expression pattern is higher related to physiological condition rather than tissue spatial distance. This result is validated by microarray data (Figure S7), although the array result is not so obvious. This may be caused by the quantitative normalization algorithm, which forces the probe intensities into the same distribution across all samples [45]–[46]. A previous hierarchical clustering of nine lungs SAGE libraries result also showed the same phenomenon with two obviously clusters of tumor and normal lung samples [42]. Regarding the biological groups of samples, gene expression pattern could be globally altered in a complex disease [45], [47]–[49]. Because the numbers of up- and down- regulated genes are roughly equal, lots of false down-regulated DE genes are produced and many up-regulated DE genes are missed [31]. This could greatly distort the biological differences between normal and cancer samples [31], but there are not any samples in different physiological condition from the same original cluster in a subgroup. In most previous studies of cancer mechanisms or other disease types, researchers often paid more attention to the differences in an individual organ under normal and disease condition. They searched for the differential expression genes to find which part of genes mainly acted to the specific tumor. Most genes provide general basic mechanisms in cancer regulation, and only a few genes play specific roles for a given cancer type. Due to the limited useful datasets our conclusion must be confirmed by abundant transcriptome sequencing.

### Human cancer signatures in gene expression

A tumor is a result of abnormal infinite cell multiplication. The comparison of normal and cancer HK gene function enrichment shows that most cancer HK genes are concerned with cell components required for cell proliferation and their corresponding biological processes (Table 2). They tend to participate in the foundational pathways that are constituted to regulate tumor occurrence, as defined by KEGG: cell cycle, pyrimidine metabolism, DNA replication, oocyte meiosis, mismatch repair, apoptosis, and so on. Our results reflect that some cancer signatures include self-sufficiency in growth signals, insensitivity to antigrowth signals, resistance to cell death, sustained angiogenesis, limitless replicative potential, instability, mutation of genome, and so on. For example, GLB1 are abundant in cancer, especially in DLD-1 cells engineered to over-express certain oncogenes [50]–[51]. TGF-b is best known for its antiproliferative effects and cancer cell evasion caused by these effects [52]–[54]. We find TGFB1 and TGFB3 gene are shown in normal HK genes group, which do not express in cancer. Cancer loses TP53 tumor suppressor function through the loss of Noxa protein expression [25]. Increasing the expression of antiapoptotic regulator Bcl2l12 in cancer can achieve similar end [25], [55]. And cancer HK genes are enriched in cell cycle and DNA replication pathways, causing limitless replicative potential. The well-known prototype of angiogenesis inhibitor thrombospondin-1 (TSP-1) [25] does not express in cancer-urged sustained angiogenesis. Cancer HK genes tend to participate in homologous recombination pathways in cancer to increase genome instability and mutation, and therefore to induce tumor emergence.

### Gene expression regulation in cancer

In RNA-Seq, there are 847 cancer-associated HK genes, while there are 2594 normal-unique HK genes. Genes are widely up-regulated and tend to be variable expression in cancer, which is confirmed by microarray data from a previous study [31]. This means a cell turns on fewer genes in cancer, but mainly up-regulates them to reply to the tumor occurrence. Variable expression regulation causes diverse cancer cell types to complete basic regulation within a smaller gene set. However, 1323 cancer-associated HK genes and 547 normal-unique HK genes were defined in selective microarray datasets from E-MTAB-27 [32] (Table S3), which are in inverse proportion to sample sets. It suffers from microarray's poor detectability and reproducibility in low-copy and transiently-expressed genes [56]. And cancer-associated HK genes' relative expression level in normal condition is higher than that in cancer condition (Table S4). This is partially caused by the normalization algorithm as previously mentioned. Otherwise, most cancer-associated HK genes in microarray data originate from shared and normal-unique HK genes defined in RNA-Seq (Table S3), in which genes tend to express higher in normal condition than in cancer condition. Microarray's weak detection of low-expressed genes may also lead to this bias.

**Figure 9:**
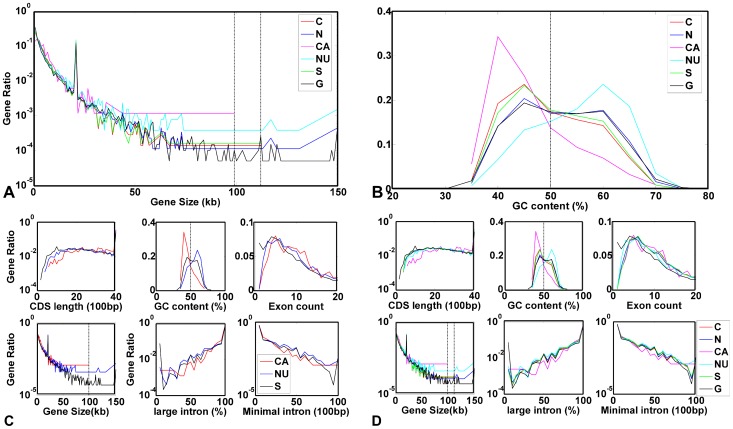
Gene structure bias among different HK gene types. Six types of gene structure are focused here, including: gene size, CDS length, number of exons, number of minimal introns, number of large introns, and GC content. We compare five types of HK genes to the background of genome (G, black line), including: cancer HK genes (C, red line), normal HK genes (N, blue line), cancer-associated HK genes (CA, purple line), normal-unique HK genes (NU, cyan line), and shared HK genes (S, green line). This reflects gene structure and sequence content affect transcription in different physiological conditions. (A) GC content bias among different HK gene types. (B) Gene size bias among different HK gene types. (C) Six types of gene structure bias in cancer-associated, normal-unique and shared HK genes, compared to the background of genome. (D) Six types of gene structure bias among five different HK gene types compared to the background of genome.

Gene structure and sequence content are considered to affect the transcriptome level. We mainly focus on gene size, CDS length, number of exons, number of minimal introns, number of large introns, and GC content. A small gene size with less large introns may enhance mRNA export from cell nucleus [14], [21], because small gene size makes gene transcribe easier. It can save energy, and more transcription can be completed. In our study, we find that cancer-associated HK genes discard large gene expression (Figure 9A) and mainly express AT-rich genes, compared to genome as background. On the other hand, normal-unique HK genes are GC-rich (Figure 9B). A GC pair is bounded by three hydrogen bonds, while an AT pair is bounded by two hydrogen bonds, which undergo breakage more easily [57]. Cancer-associated HK genes are AT-rich, which makes DNA destabilize and prone to transcribe using less energy [57]. However, CDS length, number of exons, number of minimal introns, and number of large introns weakly affect gene expression regulation (Figure 9C, D). Although alternative mRNA isoform expression varies among tissues, there is no obvious bias among alternative splitting patterns between groups (data not shown). We can infer that isoform is not a main regulated factor creates differences between two physiological conditions, normal and cancer.

## Conclusions

Gene expression pattern is higher related to physiological condition rather than tissue spatial distance. There are common regulation patterns in cancer cells, such as turn on/off regulation and low/high or constant/variable adjustment, which maintain cancer cells' limitless proliferation ability. In order to complete basic cell regulation within a smaller gene set, diverse cancer cell types turn on more widely up-regulated expression genes and turn off more constant expression genes than cells in normal condition do. Cancer-associated HK genes are enriched in cell regulation related functions and constitute some cancer signatures, including: insensitivity to antigrowth signals, resisting cell death, sustained angiogenesis, limitless replicative potential, and so on. Cancer selects AT-rich genes and discards large genes to complete cell proliferation with limited energy. These studies will help us understand the processes by which cell type-specific patterns of gene expression differ among different cell types, and particularly for cancer.

## Supporting Information

Figure S1 **RNA-Seq background threshold.**
(TIF)

Figure S2 **Illustration of tissues covered by RNA-Seq samples.** Red tissue means normal tissues that are used to identify normal HK genes. Purple and green tissues are used to validate definition of normal HK genes. Blue cell line indicates cancer cell lines that are used to define cancer HK genes. Cyan and green samples are used to validate definition of cancer HK genes. Tissue that is marked as gray colour (colorectal) contains normal and cancer sample, but they are too unsaturated to be used.
(TIF)

Figure S3 **Comparison of Daniel et.al defined ubiquitous expressed genes with our normal HK genes.** Left circle signifies Daniel et.al definition, which contains lncR (modena), unknown genes (lilac), and protein-coding genes (radiance and cyan). Right circle signifies our definition. Cyan part means protein-coding genes that overlap between them. Kelly part means protein-coding genes unique to our definition.
(TIF)

Figure S4 **Validation of defined normal HK gene in 19 normal samples and defined cancer HK gene in 13 cancer samples.** (A) Expression breadth distributions in 19 normal human tissues currently having RNA-Seq data are compared among total genes and normal HK genes defined in 12 normal tissues. Normal HK genes defined in 12 normal tissues show very broad expression in 19 tissues. (B) Expression breadth distributions in 9 cancer human cell lines currently having RNA-Seq data are compared among total genes and cancer HK genes defined in 9 normal tissues. Cancer HK genes defined in 9 cancer cell lines show very broad expression in 13 cancer samples.
(TIF)

Figure S5 **Low and high gene expression thresholds definition in the 12 normal samples.** We set a median value for low and high thresholds, respectively, in normal condition as a standard.
(TIF)

Figure S6 **Coefficient of Variation (** ***CV*** **) values distribution of normal HK genes.** The up and down bars signify Q1 (one quarter) and Q3 (three quarters) of normal HK genes' *CV* values, which are marked as constant and variable expression threshold values.
(TIF)

Figure S7 **Hierarchical cluster profiles of microarray samples based on Spearman correlation.** The Spearman correlation of gene expression profiles is used to define the expression pattern similarity of different tissues/cells from microarray samples.
(TIF)

Table S1 **Data selection and fraction of expressed HK genes in a sample.**
(DOC)

Table S2 **Microarray sample source.**
(DOC)

Table S3 **Comparison of HK gene definitions coming from RNA-Seq and microarray data.**
(DOC)

Table S4 **Cancer-associated HK genes expression comparison in normal and cancer condition from microarray data.**
(DOC)
