# Supplementary figures and images for: Identification of Human HK Genes and Gene Expression Regulation Study in Cancer from Transcriptomics Data Analysis
Source: PLoS One. 2013 Jan 31;8(1):e54082. doi: 10.1371/journal.pone.0054082 (PMC3561342; doi:10.1371/journal.pone.0054082)

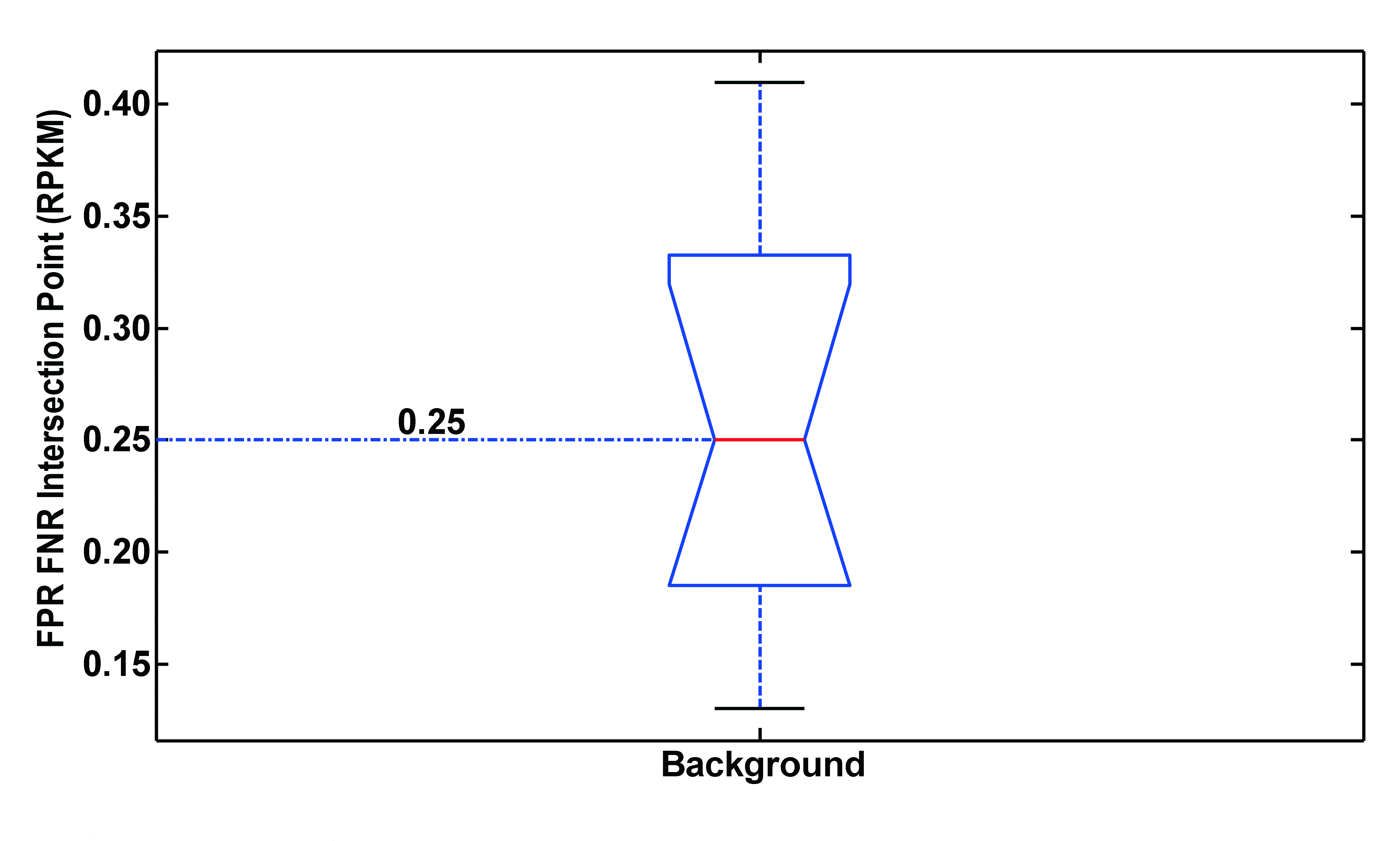

Supplement: Figure S1 — RNA-Seq background threshold. (TIF) [file pone.0054082.s001.tif]

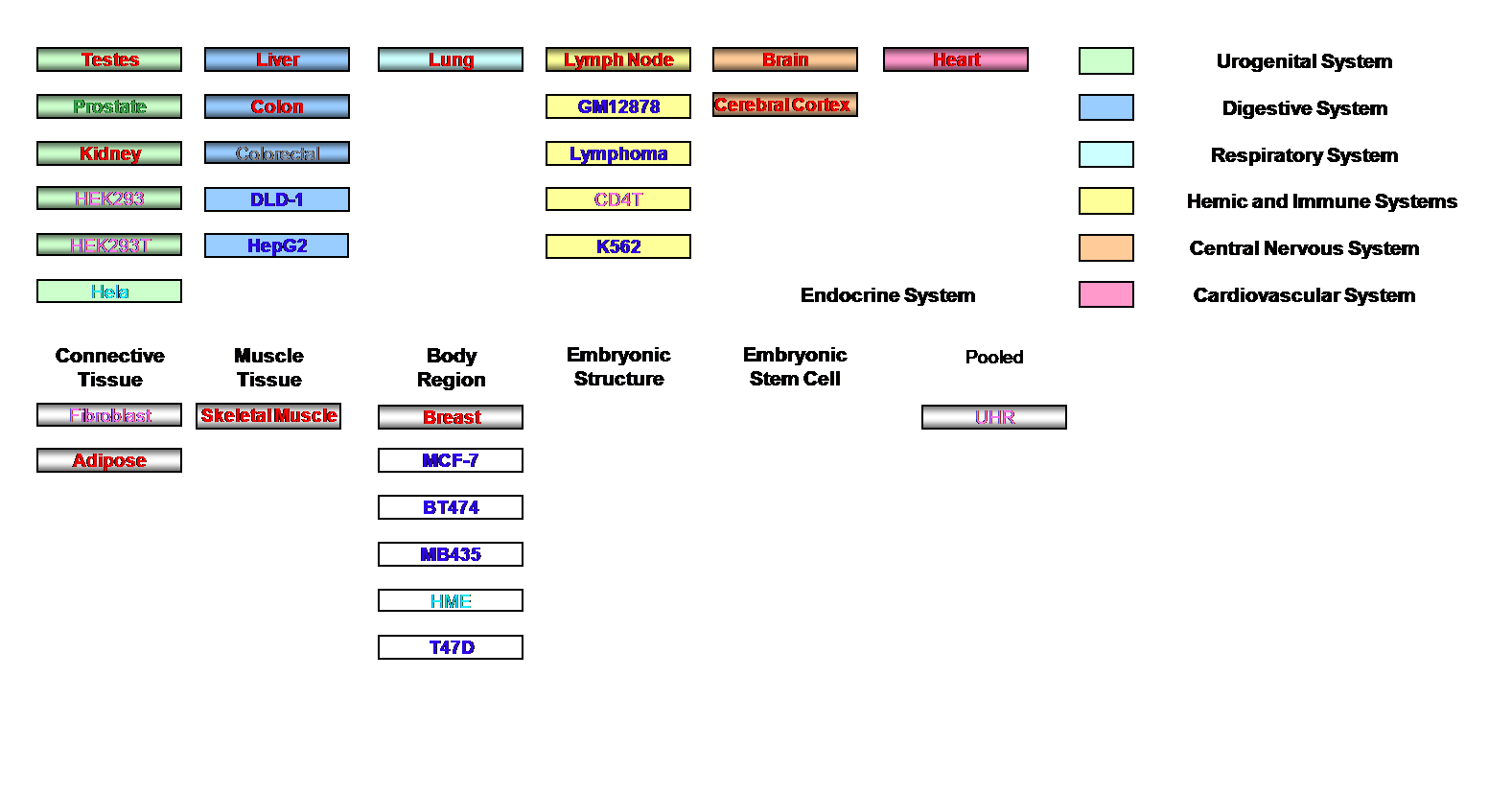

Supplement: Figure S2 — Illustration of tissues covered by RNA-Seq samples. Red tissue means normal tissues that are used to identify normal HK genes. Purple and green tissues are used to validate definition of normal HK genes. Blue cell line indicates cancer cell lines that are used to define cancer HK genes. Cyan and green samples are used to validate definition of cancer HK genes. Tissue that is marked as gray colour (colorectal) contains normal and cancer sample, but they are too unsaturated to be used. (TIF) [file pone.0054082.s002.tif]

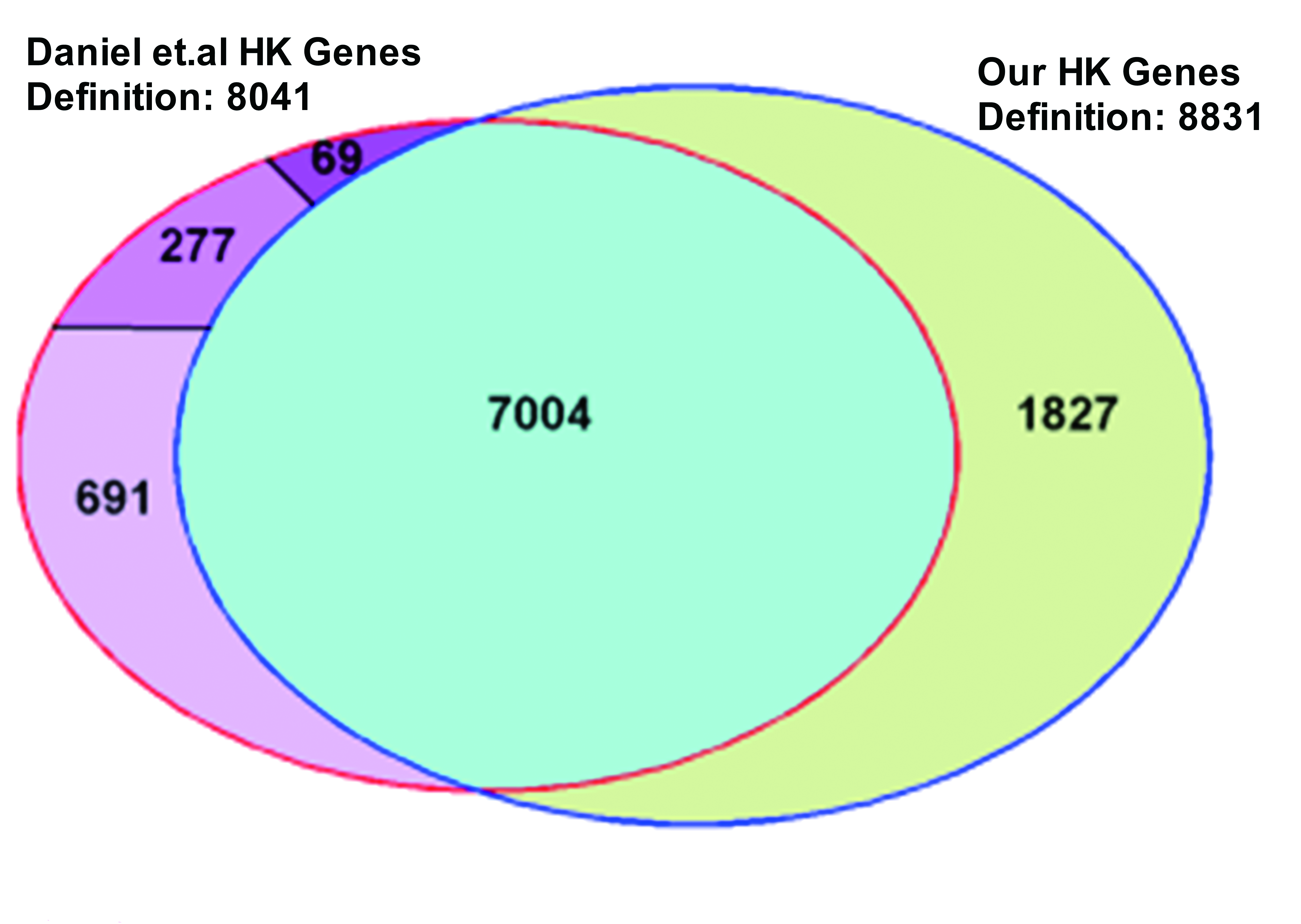

Supplement: Figure S3 — Comparison of Daniel et.al defined ubiquitous expressed genes with our normal HK genes. Left circle signifies Daniel et.al definition, which contains lncR (modena), unknown genes (lilac), and protein-coding genes (radiance and cyan). Right circle signifies our definition. Cyan part means protein-coding genes that overlap between them. Kelly part means protein-coding genes unique to our definition. (TIF) [file pone.0054082.s003.tif]

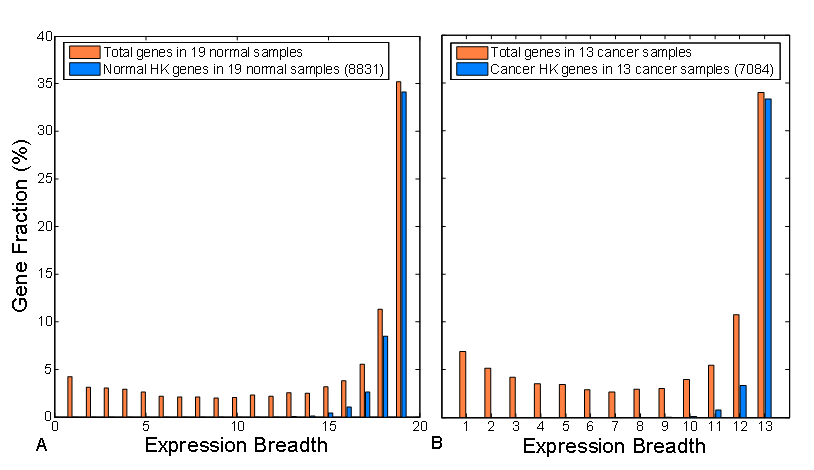

Supplement: Figure S4 — Validation of defined normal HK gene in 19 normal samples and defined cancer HK gene in 13 cancer samples. (A) Expression breadth distributions in 19 normal human tissues currently having RNA-Seq data are compared among total genes and normal HK genes defined in 12 normal tissues. Normal HK genes defined in 12 normal tissues show very broad expression in 19 tissues. (B) Expression breadth distributions in 9 cancer human cell lines currently having RNA-Seq data are compared among total genes and cancer HK genes defined in 9 normal tissues. Cancer HK genes defined in 9 cancer cell lines show very broad expression in 13 cancer samples. (TIF) [file pone.0054082.s004.tif]

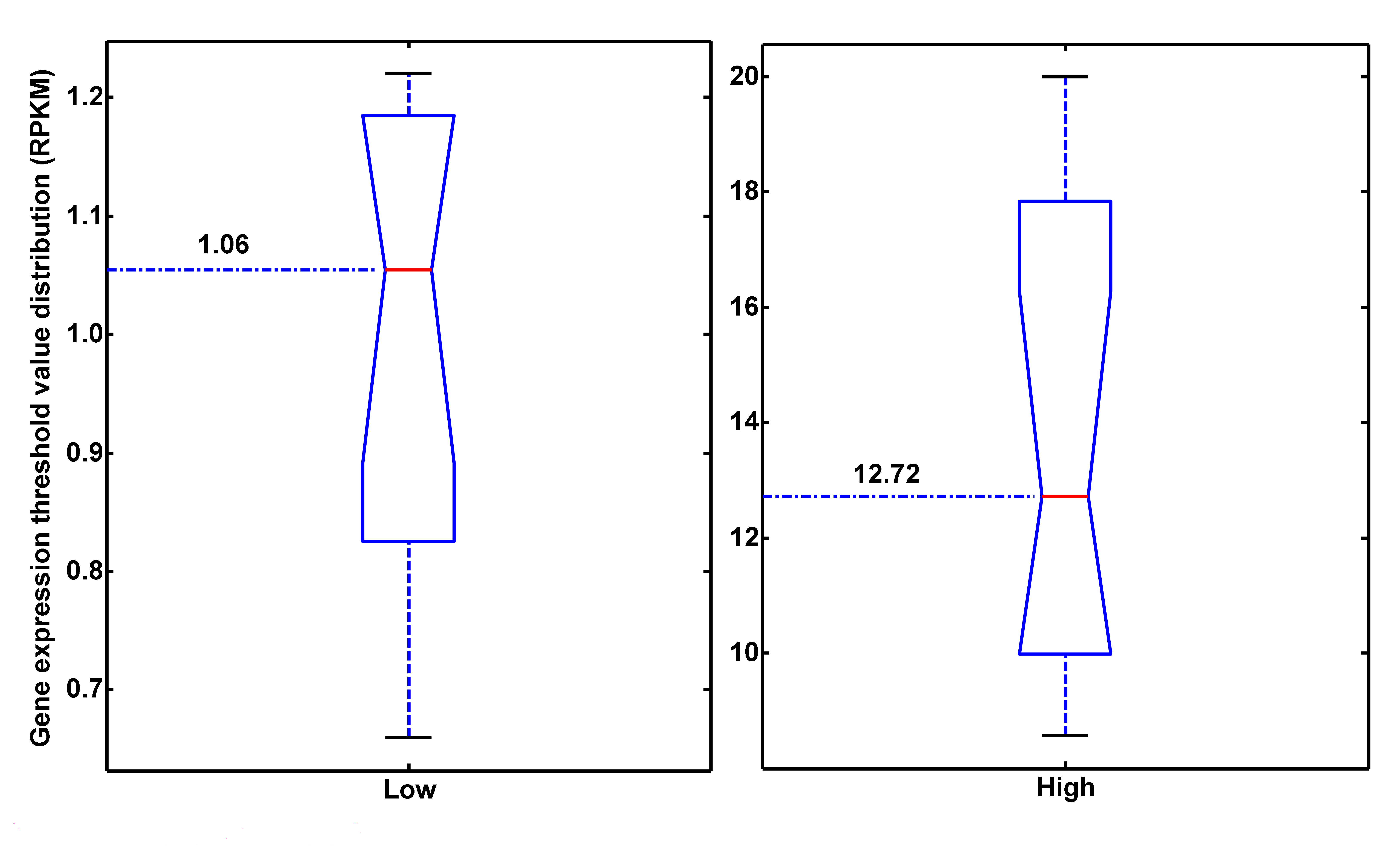

Supplement: Figure S5 — Low and high gene expression thresholds definition in the 12 normal samples. We set a median value for low and high thresholds, respectively, in normal condition as a standard. (TIF) [file pone.0054082.s005.tif]

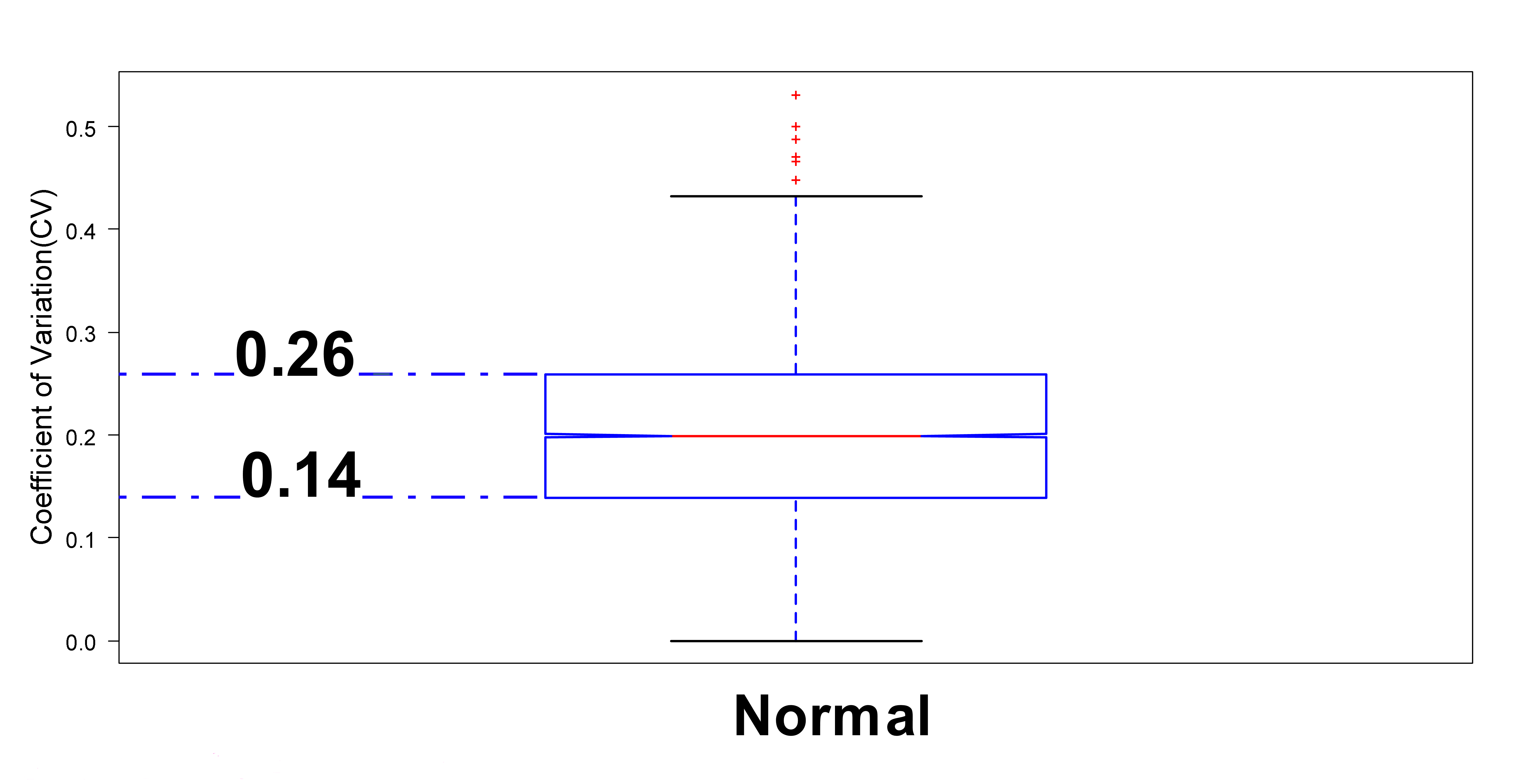

Supplement: Figure S6 — Coefficient of Variation ( CV ) values distribution of normal HK genes. The up and down bars signify Q1 (one quarter) and Q3 (three quarters) of normal HK genes' CV values, which are marked as constant and variable expression threshold values. (TIF) [file pone.0054082.s006.tif]

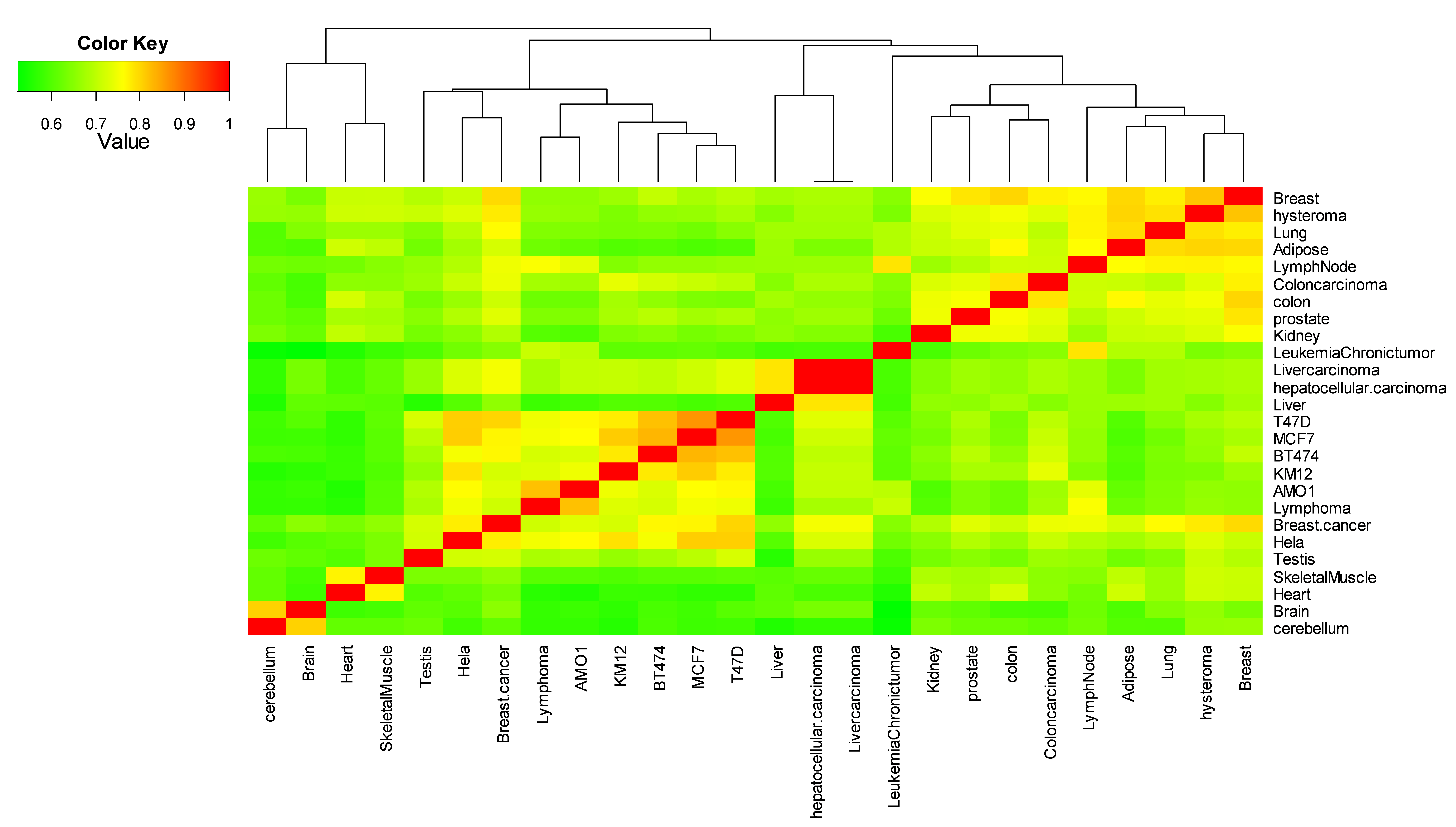

Supplement: Figure S7 — Hierarchical cluster profiles of microarray samples based on Spearman correlation. The Spearman correlation of gene expression profiles is used to define the expression pattern similarity of different tissues/cells from microarray samples. (TIF) [file pone.0054082.s007.tif]
