# Supplementary material for: Identification of Human HK Genes and Gene Expression Regulation Study in Cancer from Transcriptomics Data Analysis
Source: PLoS One. 2013 Jan 31;8(1):e54082. doi: 10.1371/journal.pone.0054082 (PMC3561342; doi:10.1371/journal.pone.0054082)
Supplement: Table S1 — Data selection and fraction of expressed HK genes in a sample. (DOC) [file pone.0054082.s008.doc]

**Table S1. Data selection and fraction of expressed HK genes in a sample.**

| **Tissue** | **SRA Accession** | **Physiological status** | **Filter reason** | **Expressed Genes** | **HK Gene Fraction§** |
| --- | --- | --- | --- | --- | --- |
| Adipose | SRS002671 | Normal |  | 12,331 | 0.72 |
| Brain | SRS002659 | Normal |  | 12,897 | 0.68 |
| Breast | SRS002661 | Normal |  | 13,004 | 0.68 |
| Cerebral Cortex | SRS002251 | Normal |  | 13,494 | 0.65 |
| Colon | SRS002670 | Normal |  | 12,681 | 0.7 |
| Heart | SRS002668 | Normal |  | 11,700 | 0.75 |
| Kidney | SRS000562 | Normal |  | 13,108 | 0.67 |
| Liver | SRS002667 | Normal |  | 11,559 | 0.76 |
| Lung | SRS002255 | Normal |  | 13,379 | 0.66 |
| Lymph Node | SRS002673 | Normal |  | 13,237 | 0.67 |
| Muscle | SRS002669 | Normal |  | 11,228 | 0.79 |
| Testis | SRS002672 | Normal |  | 15,317 | 0.58 |
| Brain cell | SRS002665 | Normal | Mixed cell lines | 14,006 | 0.63 |
| UHR | SRS002666 | Normal | Mixed cell lines | 14,210 | 0.61 |
| CD4T | SRS005031 | Normal | Special treatment | 11,553 | 0.70 |
| HEK293 | SRS001837 | Normal | Unsaturation | 9,900 | 0.72 |
| HEK293T | SRS004926 | Normal | Unsaturation | 12,152 | 0.69 |
| Fibroblasts* | SRS001833 | Normal | Significant unsaturation | 5,620 | 0.69 |
| Colorectal_rep1* | SRS210917 | Normal | Significant unsaturation | 4,140 | 0.75 |
| Colorectal_rep2* | SRS210919 | Normal | Significant unsaturation | 5,959 | 0.69 |
| Prostate_rep1 | SRS084164 | Normal | Unsaturation | 12,666 | 0.66 |
| Prostate_rep2 | SRS084170 | Normal | Unsaturation | 14,407 | 0.61 |
| BT474 | SRS002674 | Cancer |  | 11,618 | 0.61 |
| MB435 | SRS002663 | Cancer |  | 11,796 | 0.6 |
| MCF7 | SRS002662 | Cancer |  | 11,977 | 0.59 |
| T47D | SRS002664 | Cancer |  | 11,938 | 0.59 |
| DLD_1 | SRS001842 | Cancer |  | 9,345 | 0.76 |
| GM12878 | SRS000567 | Cancer |  | 11,479 | 0.62 |
| HepG2 | SRS000570 | Cancer |  | 11,613 | 0.61 |
| K562 | SRS000571 | Cancer |  | 11,407 | 0.62 |
| Lymphoma | SRS004928 | Cancer |  | 10,330 | 0.68 |
| HME | SRS002660 | Cancer | Unsaturation | 12,163 | 0.58 |
| Colorectal_rep1* | SRS210918 | Cancer | Significant unsaturation | 6,124 | 0.54 |
| Colorectal_rep2* | SRS210920 | Cancer | Significant unsaturation | 5,635 | 0.58 |
| Prostate_rep1 | SRS084144 | Cancer | Unsaturation | 12,327 | 0.54 |
| Prostate_rep2 | SRS084152 | Cancer | Unsaturation | 13,551 | 0.51 |
| Hela | Unpublished | Cancer | The only SOLiD sample | 10,252 | 0.66 |

§. In normal sample, HK gene fraction is defined as propotion of expressed normal HK genes (8831 normal HK genes) from total expressed genes in sample. In cancer sample, HK gene fraction is defined as propotion of expressed cancer HK genes (7084 cancer HK genes) from total expressed genes in sample.

*. The dataset was cancerled in our analysis for its siginificant unsaturation. There are just about 6000 genes express in a sample.
