# Supplementary material for: Identification of Human HK Genes and Gene Expression Regulation Study in Cancer from Transcriptomics Data Analysis
Source: PLoS One. 2013 Jan 31;8(1):e54082. doi: 10.1371/journal.pone.0054082 (PMC3561342; doi:10.1371/journal.pone.0054082)
Supplement: Table S2 — Microarray sample source. (DOC) [file pone.0054082.s009.doc]

**Table S2. Microarray sample source.**

| **CEL Accession** | **Pathological status** | **Sample name** |
| --- | --- | --- |
| 1229967928.CEL | cancer | MDAMB453 |
| 1229968168.CEL | cancer | BT474 |
| 586619623.CEL | cancer | T47D |
| 919973875.CEL | cancer | CervixHela |
| GSM119011.CEL | cancer | MCF7 |
| GSM95562.CEL | cancer | Lymphoma |
| 882906194.CEL | cancer | LeukemiaChronictumor |
| 557765448.CEL | cancer | hepatocellular carcinoma |
| GSM133663.CEL | cancer | KM12 |
| GSM143344.CEL | cancer | AMO-1 |
| 557765448.CEL | cancer | Livercarcinoma |
| GSM150982.CEL | cancer | Breast cancer |
| GSM38681.CEL | cancer | Hysteroma |
| GSM92276.CEL | cancer | coloncarcinoma |
| 382874628.CEL | normal | Liver |
| 391746154.CEL | normal | Lung |
| 507608494.CEL | normal | Brain |
| GSM114845.CEL | normal | Adipose |
| GSM51377.CEL | normal | LymphNode |
| GSM72495.CEL | normal | Testis |
| GSM74356.CEL | normal | SkeletalMuscle |
| GSM44689.CEL | normal | Cerebellum |
| GSM391107.CEL | normal | Kidney |
| GSM44671.CEL | normal | Heart |
| GSM44683.CEL | normal | Breast |
| GSM44680.CEL | normal | Colon |
