# Supplementary material for: Identification of Human HK Genes and Gene Expression Regulation Study in Cancer from Transcriptomics Data Analysis
Source: PLoS One. 2013 Jan 31;8(1):e54082. doi: 10.1371/journal.pone.0054082 (PMC3561342; doi:10.1371/journal.pone.0054082)
Supplement: Table S3 — Comparison of HK gene definitions coming from RNA-Seq and microarray data. (DOC) [file pone.0054082.s010.doc]

## Table S3. Comparison of HK gene definitions coming from RNA-Seq and microarray data.

|  | **Microarray** | | | | |
| --- | --- | --- | --- | --- | --- |
| **RNA-seq** | **Cancer-associated HK** | **Shared HK** | **Normal-unique HK** | **Non-HK** | **Total gene** |
| Cancer-associated HK | 139 | 79 | 14 | 615 | 847 |
| Shared HK | 985 | 2,321 | 151 | 2,780 | 6,237 |
| Normal-unique HK | 107 | 180 | 280 | 2,027 | 2,594 |
| Non-HK | 92 | 106 | 102 | -- | 300 |
| Total gene | 1,323 | 2,686 | 547 | 5,422 | 9,978 |

## 
