# Supplementary material for: Identification of Human HK Genes and Gene Expression Regulation Study in Cancer from Transcriptomics Data Analysis
Source: PLoS One. 2013 Jan 31;8(1):e54082. doi: 10.1371/journal.pone.0054082 (PMC3561342; doi:10.1371/journal.pone.0054082)
Supplement: Table S4 — Cancer-associated HK genes expression comparison in normal and cancer condition from microarray data. (DOC) [file pone.0054082.s011.doc]

## Table S4. Cancer-associated HK genes expression comparison in normal and cancer condition from microarray data.

| **Type^1^** | **T**-**test result of null hypothesis^2^** | **P**-**value** | **95% confidence interval** | **T-test statistical value** | **Degree of freedom** | **Estimated population standard deviation** |
| --- | --- | --- | --- | --- | --- | --- |
| MD | 1 | 6.16E-05 | [-0.01 -0.00] | -4.02 | 1322 | 0.09 |
| AD | 1 | 1.66E-08 | [0.01 0.02] | 5.68 | 1322 | 0.09 |

1. To characterize the discrepancy of gene expression pattern in cancer condition vs. normal condition. MD, M Distance, is defined as $MD=(\log_{10}^{\max}-\log_{10}^{\min})_{normal}-(\log_{10}^{\max}-\log_{10}^{\min})_{cancer}$; AD, A Distance, is defined as $AD=(\log_{10}^{\max}+\log_{10}^{\min})_{normal}-(\log_{10}^{\max}+\log_{10}^{\min})_{cancer}$. All cancer-associated HK genes are divided into three subtypes, constant, moderate variable (Moderate), and variable.
2. Null hypothesis is the dataset coming from a distribution with mean zero.
